# Contact Lens-Associated Ocular Surface and Corneal Disorders

**DOI:** 10.3390/mps9030095

**Published:** 2026-06-10

**Authors:** Omar Abdelaziz, Seyyedehfatemeh Ghalibafan, Raul E. Ruiz-Lozano, Jeffrey C. Peterson, Ryan A. Gallo, Ali R. Djalilian

**Affiliations:** Department of Ophthalmology and Visual Sciences, University of Illinois Chicago, 1855 W Taylor Street, Chicago, IL 60612, USA; oabdel21@uic.edu (O.A.); fatima25@uic.edu (S.G.); raule.ruiz91@gmail.com (R.E.R.-L.); jpeter56@uic.edu (J.C.P.); rgallo3@uic.edu (R.A.G.)

**Keywords:** contact lens complications, microbial keratitis, corneal infiltrates, dry eye, risk factors, patient education

## Abstract

Contact lens wear is widely used for vision correction by millions of individuals worldwide; however, it remains associated with a spectrum of ocular complications ranging from mild inflammatory conditions to vision-threatening infections. Common contact lens-related complications are predominantly noninfectious, including contact lens discomfort, dry eye syndromes, and papillary conjunctivitis. These conditions are typically mild and manageable with conservative measures. In contrast, corneal inflammatory conditions, such as contact lens-induced acute red eye and peripheral ulcers, represent an intermediate spectrum and may clinically overlap with early infection, creating diagnostic uncertainty. The most serious complication is microbial keratitis, a vision-threatening infection that remains challenging to recognize in its early stages due to its variable and often subtle presentation. Delayed identification may lead to rapid progression and significant visual morbidity. Patients with contact lens-related complaints often present to frontline settings, where early recognition is essential. Distinguishing benign from infectious conditions can be challenging; a risk-based approach with prompt triage and referral, along with proper lens hygiene and patient education, is key.

## 1. Introduction

Contact lenses provide safe and effective vision correction for approximately 45 million individuals in the United States and an estimated 125 million wearers worldwide [1,2]. Their clinical value extends beyond optical correction by improving functional vision and quality of life for many wearers. However, contact lens use may also disrupt ocular surface homeostasis and is associated with a spectrum of complications ranging from mild discomfort to vision-threatening infections [3,4].

Soft contact lenses account for most contact lens wear and the majority of reported contact lens-related complications. In contrast, rigid gas-permeable and scleral lenses possess distinct complication profiles that may include mechanical irritation, epithelial staining, corneal warpage, hypoxia-related changes, conjunctival compression, midday fogging, and fitting-related problems [3,5].

Approximately 1 million individuals in the United States seek medical care for contact lens-related complications annually, accounting for an estimated $175 million in direct healthcare expenditures [1,6,7]. These data underscore the substantial public health burden of contact lens complications and highlight the importance of prevention, timely identification, and appropriate management.

Primary care providers occupy a critical role in the prevention and early detection of contact lens-related complications. As the first point of contact for many patients presenting with ocular symptoms, they are often required to distinguish between benign and potentially vision-threatening conditions [8,9]. However, this responsibility is frequently challenged by limited access to specialized ophthalmic equipment and the absence of clear, standardized guidance for evaluation and management [9].

The largely preventable nature of most contact lens complications further emphasizes the importance of primary care involvement [10]. Population-based surveys indicate that up to 99% of contact lens wearers report at least one hygiene-related risk behavior, such as overnight wear, improper replacement of disinfecting solutions, or exposure of lenses to tap water [1,9,10]. In addition, nearly one-third of wearers do not recall receiving adequate instruction on lens care from healthcare providers [4,11]. These findings highlight a significant opportunity to improve patient education during routine clinical encounters.

Common contact lens-related complications include discomfort and dry eye syndromes, papillary conjunctivitis, and corneal infiltrative events. In contrast, microbial keratitis—although less frequent—represents the most serious complication, with an estimated annual incidence of 2–4 cases per 10,000 contact lens wearers [3,12,13]. Analysis of FDA adverse event reports from 2005 to 2015 demonstrated that nearly one in five contact lens-related infections (19.8%) resulted in serious outcomes, including central corneal scarring, reduced visual acuity, or the need for corneal transplantation, underscoring the importance of prevention and timely management [6].

This review provides an evidence-based framework for evaluating and managing contact lens complications, emphasizing clinical presentation, risk stratification, triage, patient education, and appropriate referral.

## 2. Ocular Surface and Tear Film-Related Disorders

Most contact lens-related complications encountered in primary care involve the ocular surface and tear film and are generally noninfectious and non-vision-threatening, allowing for conservative management. These conditions share overlapping mechanisms, including tear film instability, mechanical irritation, and low-grade inflammation [14,15,16,17].

Contact lens discomfort (CLD), along with contact lens-associated dry eye (CLADE) and contact lens-induced dry eye (CLIDE), represent the most common complications of contact lens wear. CLD affects approximately 35–58% of wearers and is a leading cause of contact lens discontinuation [16,17,18,19,20]. Although its underlying pathophysiology remains incompletely understood, tools such as the Contact Lens Discomfort Index (CLDI) may assist in clinical assessment [21,22].

CLADE refers to persistent dry eye symptoms in individuals with preexisting ocular surface disease, whereas CLIDE symptoms resolve with discontinuation of lens wear [3]. These conditions are highly prevalent, with up to 70% of contact lens users reporting mild-to-severe symptoms and approximately 25% experiencing severe disease based on Ocular Surface Disease Index (OSDI) scores [23]. They are associated with tear film disruption, increased friction between the lens and ocular surface, and, in some cases, changes in tear biomarkers [24,25,26,27].

Clinically, patients typically present with dryness, burning, foreign body sensation, fluctuating vision, and increased lens awareness, often worsening with prolonged wear and toward the end of the day [3,18]. Risk factors include prolonged wear, high-water content lenses, poor hygiene, coexisting ocular surface disease, and environmental exposures such as wind or air conditioning [3,19,23].

## 3. Conjunctival Inflammatory Disorders

Contact lens-induced papillary conjunctivitis (CLPC), also referred to as giant papillary conjunctivitis, is a common inflammatory condition involving the superior tarsal conjunctiva. It results from a combination of mechanical irritation and immune responses to protein deposits on the lens surface, with reported incidence reaching up to 47.5% depending on lens type and wear modality [28,29,30,31].

CLPC typically develops after a period of previously successful lens wear and is associated with risk factors such as prolonged wear, infrequent lens replacement, inadequate hygiene, and atopy [29]. Patients commonly present with itching, mucus discharge, redness, blurred vision, and reduced lens tolerance [30]. Histopathologic findings include increased conjunctival mast cells, eosinophils, and basophils [30,32]. Although inflammatory, CLPC is confined to the conjunctiva and is generally non-vision-threatening.

## 4. Corneal Inflammatory Conditions

Corneal complications represent a more clinically significant group of contact lens-related disorders, as they involve the cornea and may overlap with early infection [33,34]. These conditions are typically driven by inflammatory responses to bacterial antigens, mechanical irritation, or hypoxia, and may clinically resemble early microbial keratitis, making accurate triage essential in the primary care setting [35].

### 4.1. Corneal Infiltrative Events

Corneal infiltrative events (CIEs) encompass a spectrum of inflammatory corneal responses characterized by corneal infiltrates with or without epithelial involvement [35]. Although often sterile, their clinical presentation may mimic early infection, necessitating careful evaluation and follow-up [35].

### 4.2. Contact Lens-Induced Acute Red Eye and Peripheral Ulcer

Contact lens-induced acute red eye (CLARE) is an acute inflammatory condition associated with overnight or extended wear [36,37]. Patients present with sudden-onset redness, mild-to-moderate discomfort, tearing, and photophobia, often noted upon awakening [38]. This condition reflects a sterile inflammatory response to bacterial colonization and endotoxin release rather than true infection [38].

Contact lens-induced peripheral ulcer (CLPU) is a localized inflammatory lesion characterized by a small peripheral epithelial defect with underlying stromal infiltration [39,40]. It is thought to result from bacterial toxins in the setting of epithelial disruption [41,42]. Patients present with mild-to-moderate pain and peripheral corneal involvement, with relatively rapid improvement after discontinuation of lens wear [43].

Risk factors include overnight wear, microbial contamination (particularly *Staphylococcus aureus* and *Pseudomonas* species), tight-fitting lenses, and the contact lens-associated epithelial trauma [41]. However, distinguishing these conditions from microbial keratitis remains challenging. In one study, only 20% of cases could be definitively classified, while over half demonstrated overlapping features [43,44]. Clinicians should therefore maintain a low threshold for urgent ophthalmology referrals when symptoms are severe, vision is reduced, or findings are concerning.

Inflammation contributes to several contact lens-associated ocular surface and corneal disorders [21,26,27]. Mechanical friction, lens deposits, hypoxia, tear film instability, and microbial products may activate epithelial and immune pathways, leading to cytokine release, inflammatory cell recruitment, and disruption of ocular surface homeostasis [21,26,37]. In milder cases, these responses may present as discomfort, dryness, papillary conjunctivitis, or sterile corneal infiltrative events [3,29,38]. However, similar inflammatory findings may also accompany early infection, contributing to the clinical overlap between noninfectious and infectious presentations [38,43,44].

## 5. Infectious Corneal Disease: Microbial Keratitis

Microbial keratitis represents the most serious contact lens-related complication and is characterized by infection of the corneal epithelium and stroma by bacterial, fungal, or parasitic organisms [5,41]. The overall incidence has varied by lens type and wear modality [45,46,47].

The pathogenesis involves corneal hypoxia, mechanical epithelial disruption, and microbial contamination of lenses or lens care solutions [48,49]. Direct inoculation may also occur through poor hand hygiene. Hypoxia enhances susceptibility to *P. aeruginosa* infection by increasing bacterial adherence and modulating host defense pathways, including cystic fibrosis transmembrane conductance regulator (CFTR) and NF-κB signaling [50]. Mechanical epithelial disruption further compromises the epithelial barrier [51].

Clinically, patients present with eye pain, photophobia, decreased vision, purulent discharge, and conjunctival injection [44]. However, patients may also present with subtle symptoms that mimic benign conditions (e.g., CLD, CLIDE, CLADE), thus delaying diagnosis and treatment. Examination may reveal epithelial defects, stromal infiltration, corneal edema, and hypopyon [44]. Bacterial keratitis typically presents acutely within 24–48 h, whereas *Acanthamoeba* keratitis has a more insidious onset and is frequently misdiagnosed [52,53,54]. Delayed diagnosis of Acanthamoeba keratitis is associated with poorer visual outcomes, prolonged treatment courses, progressive stromal involvement, corneal scarring, and an increased need for surgical intervention, including therapeutic or optical keratoplasty [53,55,56]. Severe or refractory cases may progress to corneal perforation, require repeat transplantation, or, rarely, require keratoprosthesis or globe-removing procedures, underscoring the importance of early recognition and targeted therapy [55,57]. Fungal keratitis generally follows a chronic course with characteristic feathery infiltrates [58,59].

Risk factors include overnight or extended wear, water exposure, poor hygiene practices, and failure to adhere to recommended replacement schedules [1]. Exceeding replacement intervals significantly increases risk, and even occasional overnight wear has been associated with a markedly elevated risk of microbial keratitis [47].

**Figure 1:**
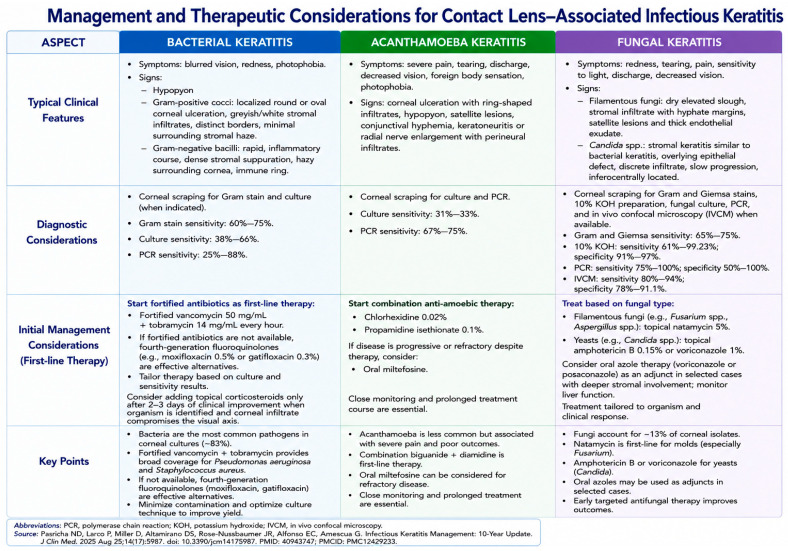
Initial Management Considerations for Contact Lens-Associated Infectious Keratitis. Comparison of the typical clinical features, diagnostic considerations, first-line therapeutic approaches, and key clinical considerations for bacterial, Acanthamoeba, and fungal keratitis in contact lens wearers. Information compiled and synthesized from published literature, including Pasricha et al. [56].

Key clinical features, diagnostic considerations, and initial management approaches for major forms of contact lens-associated infectious keratitis are summarized in Figure 1.

## 6. Primary Care Evaluation and Triage

### 6.1. History and Risk Assessment

The primary goal of evaluation is to identify features suggestive of infection and determine the need for urgent referral. A focused history is essential to differentiate between benign and vision-threatening conditions. Relevant patient-related factors include prior ocular infections, history of trauma, known eye disorders, diabetes, autoimmune disease, past ocular surgeries, medication use (e.g., corticosteroids or immunomodulators), and allergies [41].

A detailed contact lens history is essential, encompassing lens type (daily disposable, biweekly, monthly, rigid gas permeable, soft, or scleral), duration and wearing schedule, as well as high-risk exposures such as overnight use, water contact (swimming, showering, hot tubs), and recent changes in care products [60,61,62,63]. Lens hygiene practices should be carefully assessed, including cleaning routine, solution type, frequency, and storage habits (case hygiene and replacement), with particular attention to high-risk behaviors such as tap water use and “topping off” solution, which increase the risk of microbial contamination and infectious keratitis; adherence to recommended replacement schedules should also be reviewed [64,65].

**Figure 2:**
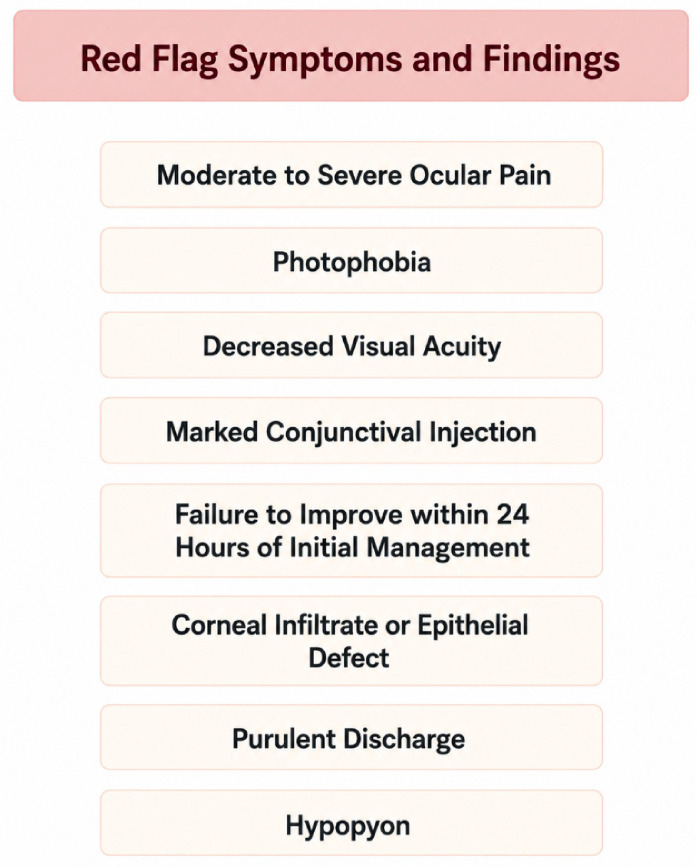
Red Flags in Contact Lens Wearers Requiring Ophthalmology Referral.

Symptom assessment includes onset, duration, progression, severity, and relationship to contact lens wear. Active screening for red flag features—such as moderate to severe ocular pain, photophobia, decreased visual acuity, marked conjunctival injection, or lack of improvement within 24 h—is essential [65] (Figure 2).

### 6.2. Ocular Examination

Visual acuity is assessed in both eyes whenever possible, as it remains a key component of the ocular examination [44]. External inspection includes evaluation for conjunctival injection, discharge, eyelid edema, and gross corneal opacity, along with assessment of pupillary responses and extraocular movements [63,66]. When available, fluorescein staining is used to identify corneal epithelial erosions and defects [43,44].

If a corneal infiltrate or epithelial defect is identified, differentiation between inflammatory corneal events and suspected microbial keratitis becomes essential. Small peripheral infiltrates without a significant epithelial defect are more commonly associated with inflammatory conditions such as CLARE or CLPU [43]. Features favoring a noninfectious process include peripheral location, small infiltrate size (≤1–2 mm), minimal or absent epithelial defect, mild to moderate discomfort, absence of mucopurulent discharge, and rapid improvement following discontinuation of contact lens wear [43] (Table 1).

However, early microbial keratitis may initially resemble these inflammatory presentations. Clinicians should therefore maintain a low threshold for urgent ophthalmology referral when there is significant pain, reduced vision, central or paracentral involvement, a prominent epithelial defect, purulent discharge, or diagnostic uncertainty [39,67].

### 6.3. Initial Management and Referral

For all patients with suspected contact lens-related complications, contact lens wear is discontinued until symptoms resolve and an appropriate diagnosis is established [41]. Topical corticosteroids should not be initiated empirically in the primary care setting when microbial keratitis remains within the differential diagnosis. Although corticosteroids may have a role in selected cases of infectious keratitis after appropriate antimicrobial therapy has been established and under specialist supervision, their use depends on the causative pathogen, stage of disease, epithelial status, and clinical response. Premature or unsupervised corticosteroid use may worsen infection, delay epithelial healing, and mask clinical progression [68]. Eye patching is also avoided due to the risk of creating a warm, occlusive environment that promotes microbial growth [69]. Topical anesthetics are not prescribed for outpatient use, as they impair epithelial healing and may mask disease progression [70].

Patients with suspected microbial keratitis or CLPU require urgent ophthalmology referral [67,71]. When immediate ophthalmologic evaluation is not available, empiric broad-spectrum topical antibiotic therapy may be initiated while arranging referral. Broad-spectrum topical antibiotics remain the mainstay of initial treatment for bacterial keratitis, with topical fluoroquinolones commonly used in this setting [72].

In the absence of a corneal infiltrate or epithelial defect, noninfectious contact lens-related conditions such as CLADE, CLIDE, CLPC, or CLD are considered. These conditions typically present with dryness, burning, itching, foreign body sensation, mild conjunctival injection, and reduced contact lens tolerance, with minimal pain and little or no visual disturbance [19]. Initial management includes temporary discontinuation of contact lens wear, preservative-free artificial tears, optimization of lens hygiene, and, when CLPC is suspected, antihistamine or mast cell-stabilizing therapy [73,74,75,76].

Artificial tears are available in various formulations, including hydroxypropyl guar, hyaluronic acid, and lipid-based nanoemulsions [77,78]. In contact lens wearers, twice-daily application—10 min before insertion and after removal—has been shown to improve comfort and reduce dryness [79].

Patients with persistent symptoms despite conservative therapy are referred to an eye care specialist for further evaluation [41].

**Figure 3:**
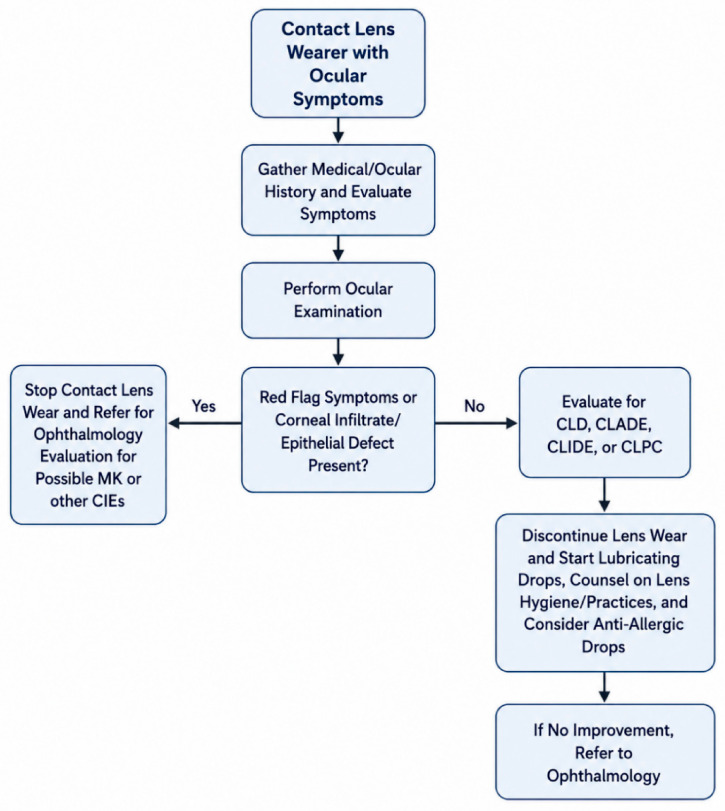
Triage Algorithm for Contact Lens Complications in Primary Care Setting.

Table 2 summarizes common noninfectious conditions and their management, and Figure 3 outlines a triage algorithm for clinical decision-making.

## 7. Patient Education and Prevention in Primary Care

### 7.1. Contact Lens Hygiene Counseling

Because most contact lens-related complications arise from modifiable behaviors, patient education represents one of the most effective preventive strategies. Population-based surveys indicate that nearly all contact lens wearers report at least one hygiene-related risk behavior, including sleeping in lenses, improper solution use, and exposure to water sources [1,10]. Despite this, many patients report receiving limited counseling from healthcare providers [11,80].

Primary care clinicians play an important role in reinforcing fundamental hygiene practices during routine encounters. Proper lens care includes washing and drying hands before handling lenses, replacing disinfecting solution daily rather than “topping off,” and regularly cleaning and replacing contact lens storage cases [81]. Cases are air-dried between uses and replaced at least every three months to reduce microbial contamination [81]. Regular case replacement has been shown to be strongly protective against infection (OR = 0.14, *p* < 0.001) [82].

### 7.2. Behavioral Risk Reduction

Water exposure is a major risk factor for microbial keratitis. Contact lenses should not be worn while swimming, showering, or using hot tubs due to exposure to organisms such as *Acanthamoeba* [17]. Showering in contact lenses has been associated with a significantly increased risk of microbial keratitis (OR = 3.1, *p* = 0.03) [83]. Rinsing lenses or storage cases with tap water is also avoided. When water exposure is unavoidable, daily disposable lenses may be considered to reduce contamination risk [82].

Overnight or extended wear remains one of the strongest risk factors for microbial keratitis and is avoided unless specifically prescribed [83]. Lenses used during episodes of ocular irritation are discarded, and sharing of lenses or accessories is avoided. Regular follow-up with an eye care professional is recommended to monitor ocular surface health and detect early complications [65].

### 7.3. Recognition of Warning Symptoms

Patient education should extend beyond general hygiene counseling to include recognition of symptom patterns that may indicate progression beyond routine lens intolerance [75]. Wearers should be advised that persistent redness, increasing discomfort, photophobia, discharge, visual change, or symptoms following water exposure require immediate discontinuation of lens wear and prompt clinical evaluation [75]. Reinforcing these warning signs during lens fitting, routine follow-up, and primary care visits may improve patient awareness and support earlier presentation before irreversible corneal damage occurs [75].

## 8. Discussion

**Figure 4:**
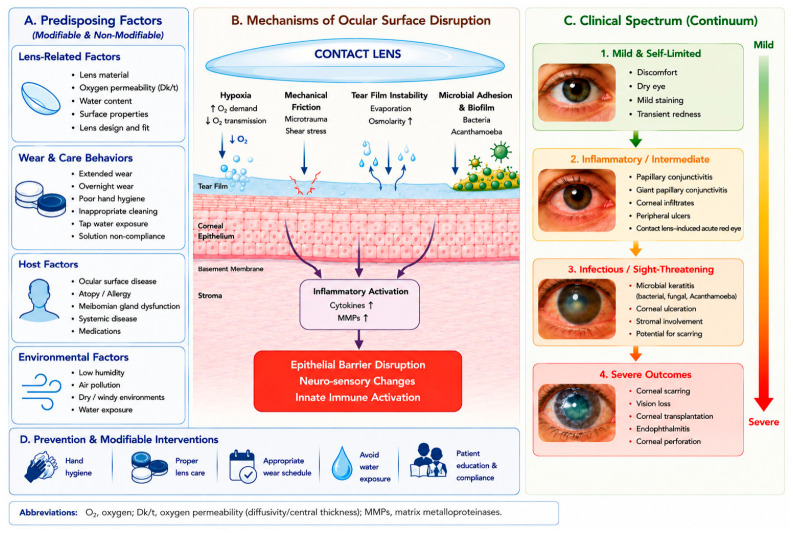
Pathophysiologic Framework and Clinical Continuum of Contact Lens-Associated Disorders. Contact lens wear may disrupt ocular surface homeostasis through interactions among lens-related characteristics, host susceptibility, hygiene behaviors, and environmental exposures. These factors contribute to hypoxia, mechanical stress, tear film instability, microbial adhesion, and inflammatory activation, resulting in a clinical spectrum from mild discomfort and dry eye symptoms to inflammatory corneal events, microbial keratitis, and severe outcomes. Preventive measures and early recognition remain critical for reducing disease burden and preserving ocular surface health.

Contact lens-related ocular disease reflects a multifactorial disruption of ocular surface homeostasis, driven by the interaction of mechanical stress, tear film instability, and microbial exposure [84]. These factors do not act independently but influence one another, producing a spectrum of responses that range from subclinical alterations to overt pathology [85]. This continuum-based behavior explains the variability in clinical expression and the frequent overlap between different disease states [86]. The multifactorial mechanisms and clinical continuum underlying contact lens-associated disorders are summarized in Figure 4.

At the tissue level, repeated contact lens wear introduces cumulative epithelial stress that may alter barrier integrity and local immune function [85]. Even in the absence of clinically apparent disease, low-grade inflammatory activity and microstructural changes can persist, modifying the ocular surface environment [87]. These alterations may lower the threshold for subsequent injury or infection, suggesting that disease development often evolves from progressive biological changes rather than isolated acute events [88].

Differences in individual susceptibility further contribute to the heterogeneity of outcomes observed among contact lens users [88]. Variations in tear film composition, epithelial resilience, and immune responsiveness may influence how the ocular surface reacts to similar external exposures [87]. Such heterogeneity highlights the importance of intrinsic host factors in shaping disease expression, beyond the influence of lens characteristics or usage patterns alone [84].

Sex-related factors may also contribute to variability in contact lens-associated disease. Contact lens wear is often more prevalent among women, and female contact lens wearers may report higher rates of dryness and discomfort [10,18,20]. Sex and hormonal factors are also recognized contributors to ocular surface and dry eye disease, which may influence symptom burden in contact lens users [89,90]. However, available evidence remains insufficient to define consistent sex-specific risk profiles across individual contact lens-associated complications, highlighting the need for further studies across different disorders.

Environmental and behavioral factors also interact with these biological processes, introducing additional variability in disease development [91]. Exposure to external contaminants, fluctuations in environmental conditions, and inconsistencies in lens use patterns can modify the ocular surface microenvironment and influence disease trajectory [91]. These dynamic interactions underscore the complexity of contact lens-related complications, arising from the continuous interplay among host, device, and environment [92].

Beyond these factors, the physical and material properties of contact lenses introduce an additional layer of influence on ocular surface dynamics. Lens modulus, oxygen permeability, surface wettability, and edge design can alter mechanical interactions with the corneal epithelium and tear film distribution during blinking [85,93]. These properties may affect not only comfort but also microenvironmental stability, including tear film renewal and debris clearance [94]. Subtle variations in these parameters can contribute to localized areas of stress or stagnation, potentially predisposing the ocular surface to inflammatory responses or microbial adherence even in otherwise compliant users [95].

## 9. Conclusions

Contact lens use is widespread and continues to increase globally, making contact lens-related complications a common presentation in clinical practice. The majority of contact lens-related complications are noninfectious and can be effectively managed with conservative measures, including temporary discontinuation of lens wear, lubrication, and reinforcement of appropriate hygiene practices. Because most complications arise from modifiable behaviors, prevention and patient education during routine clinical encounters remain essential strategies for reducing disease burden.

A key challenge, however, lies in the early recognition of infectious keratitis. Addressing this challenge requires repeated, consistent messaging at the point of lens fitting, routine follow-up visits, and general healthcare encounters, with clear emphasis on discontinuing lens wear and seeking prompt evaluation when pain, photophobia, reduced vision, persistent redness, purulent discharge, or water exposure occurs. Optometrists and ophthalmologists can reinforce these messages through standardized hygiene counseling, written safety instructions, and reminder-based education to improve awareness among contact lens wearers. This condition may initially present with subtle symptoms and be misinterpreted as a benign ocular surface disorder, leading to delayed evaluation and progression to corneal ulceration and irreversible vision loss. Therefore, contact lens-associated disorders should be viewed as a continuum of ocular surface responses shaped by lens properties, host susceptibility, hygiene behaviors, and environmental exposures, linking common conditions such as discomfort and dry eye with less frequent but severe infectious complications. This framework highlights the need for balanced contact lens use, preserving the visual and functional benefits of lenses while minimizing preventable ocular surface morbidity.

## 10. Future Directions

Future advances in contact lens-related ocular disease will depend on improving early biological characterization and developing strategies that address underlying mechanisms rather than late clinical manifestations [96,97]. Beyond conventional refractive correction, contact lenses are increasingly being used for pediatric myopia control, including orthokeratology and multifocal or dual-focus soft contact lenses [98,99]. In parallel, therapeutic contact lenses are being investigated as platforms for sustained ocular drug delivery, including antimicrobial, anti-inflammatory, and lubricating therapies for ocular infections, dry eye disease, and other ocular surface disorders [100,101]. Emerging research focused on tear film biomarkers, epithelial barrier integrity, and inflammatory signaling may enable the detection of subclinical changes preceding overt disease, allowing for earlier and more precise intervention [96,97]. In parallel, advances in high-resolution ocular surface imaging and molecular diagnostics may provide objective tools for identifying early microstructural and biochemical alterations that are not detectable through routine clinical examination [96,97]. At the same time, continued innovation in contact lens materials—particularly those designed to enhance biocompatibility, reduce microbial adhesion, and maintain tear film stability—offers the potential to mitigate key drivers of disease at their source [102,103]. Emerging material technologies are also being developed to improve contact lens comfort and wearer tolerance. These include glycopolymer-based surface modifications, biomimetic coatings, and advanced hydrogel formulations designed to enhance wettability, reduce friction, stabilize the tear film, and limit protein or microbial deposition on the lens surface [104,105]. Future developments may also incorporate responsive or “smart” materials capable of adapting to the ocular surface environment or delivering therapeutic agents in a controlled manner [101,106]. Expanding insights into ocular surface microbiome dynamics and host–environment interactions may further refine risk stratification and support more individualized approaches to contact lens use [92]. Integration of these biological, technological, and material advances will be essential for enabling a more predictive and personalized framework, to improve long-term ocular surface health and reduce variability in clinical outcomes.

## Figures and Tables

**Table 1:** Differentiating Sterile Corneal Infiltrates from Microbial Keratitis in Contact Lens Wearers [39,43,44].

Feature	Sterile Corneal Infiltrate Event (CLPU)	Microbial Keratitis
Pain	Mild to moderate	Severe
Location	Peripheral	Central or paracentral
Discharge	Minimal	Often purulent
Vision	Usually preserved	Often decreased
Epithelial defect	Minimal, small, or absent	Prominent
Course	Improves quickly	Progressive

Clinical differentiation between sterile corneal infiltrative events, particularly contact lens-induced peripheral ulcer, and early microbial keratitis may be challenging because overlapping clinical features are common. Therefore, cases with severe pain, reduced vision, central or paracentral involvement, a prominent epithelial defect, purulent discharge, progression, or diagnostic uncertainty should be managed cautiously, with a low threshold for urgent ophthalmic evaluation [39,43,44].

**Table 2:** Common Noninfectious Contact Lens-Related Conditions Encountered in Primary Care [3,19,29,38,41].

Condition	Typical Features	Primary Care Management
Contact Lens Discomfort	Dryness, foreign body sensation, burning, lens awareness, and symptoms typically intensifying toward the end of the day	Artificial tears, lens hygiene counseling
Contact Lens-Associated Dry Eye Disease (CLADE)	Dryness, burning, end-of-day discomfort, reduced contact lens tolerance, symptoms persist after contact lens removal due to underlying dry eye disease	Artificial tears, lens hygiene counseling
Contact Lens-Induced Dry Eye (CLIDE)	Dryness, burning, end-of-day discomfort, reduced contact lens tolerance, symptoms improve or resolve after contact lens removal	Artificial tears, lens hygiene counseling
Contact Lens-Induced Papillary Conjunctivitis (CLPC)	Itching, excessive mucus production, blurred vision, decreased contact lens tolerance; large papillae of the superior tarsal conjunctiva	Stop lenses 2–4 weeks, antihistamine/mast-cell stabilizer, improve lens hygiene and consider switching to daily disposable lenses
Contact Lens-Induced Acute Red Eye (CLARE)	Acute unilateral or bilateral red eye, discomfort, tearing, photophobia, often after overnight or extended lens wear; peripheral corneal infiltrates may be present	Stop lenses, preservative-free artificial tears, ophthalmology referral if unsure

The summarized clinical features and management considerations are based on prior reviews and clinical guidance regarding contact lens discomfort, contact lens-associated dry eye, contact lens-induced dry eye, contact lens-induced papillary conjunctivitis, and contact lens-induced acute red eye [3,19,29,38,41]. Management recommendations are further supported by studies and reviews addressing artificial tears and treatment approaches for dry eye disease and papillary conjunctivitis [73,74,75,76].

## Data Availability

No new data were created or analyzed in this study.

## Abbreviations

CLADE	Contact Lens-Associated Dry Eye
CLIDE	Contact Lens-Induced Dry Eye
CLD	Contact Lens Discomfort
CLDI	Contact Lens Discomfort Index
CLPC	Contact Lens-Induced Papillary Conjunctivitis
GPC	Giant Papillary Conjunctivitis
CLARE	Contact Lens-Induced Acute Red Eye
CLPU	Contact Lens-Induced Peripheral Ulcer
OSDI	Ocular Surface Disease Index
CIE	Corneal Infiltrative Event
MK	Microbial Keratitis
CFTR	Cystic Fibrosis Transmembrane Conductance Regulator
NF-κB	Nuclear Factor-κB
PCR	Polymerase Chain Reaction
KOH	Potassium Hydroxide
IVCM	In Vivo Confocal Microscopy
MMPs	Matrix Metalloproteinases
Dk/t	Oxygen Permeability
O_2_	Oxygen
OR	Odds Ratio
